# Climate change impacts on irrigated rice and wheat production in Gomti River basin of India: a case study

**DOI:** 10.1186/s40064-016-2905-y

**Published:** 2016-08-03

**Authors:** N. S. Abeysingha, Man Singh, Adlul Islam, V. K. Sehgal

**Affiliations:** 1 Department of Agricultural Engineering and Soil Science, Faculty of Agriculture, Rajarata University of Sri Lanka, Puliyankulama, Anuradhapura, Sri Lanka; 2 Water Technology Centre, Indian Agricultural Research Institute, New Delhi, 110012 India; 3 Natural Resource Management Division, KAB II, Indian Council of Agricultural Research, New Delhi, 110012 India; 4 Division of Agricultural Physics, Indian Agricultural Research Institute, New Delhi, 110012 India

**Keywords:** Climate change, Gomti River basin, Irrigation, Rice, SWAT, Wheat

## Abstract

Potential future impacts of climate change on irrigated rice and wheat production and their evapotranspiration and irrigation requirements in the Gomti River basin were assessed by integrating a widely used hydrological model “Soil and Water Assessment Tool (SWAT)” and climate change scenario generated from MIROC (HiRes) global climate model. SWAT model was calibrated and validated using monthly streamflow data of four spatially distributed gauging stations and district wise wheat and rice yields data for the districts located within the basin. Simulation results showed an increase in mean annual rice yield in the range of 5.5–6.7, 16.6–20.2 and 26–33.4 % during 2020s, 2050s and 2080s, respectively. Similarly, mean annual wheat yield is also likely to increase by 13.9–15.4, 23.6–25.6 and 25.2–27.9 % for the same future time periods. Evapotranspiration for both wheat and rice is projected to increase in the range of 3–9.6 and 7.8–16.3 %, respectively. With increase in rainfall during rice growing season, irrigation water allocation for rice is likely to decrease (<5 %) in future periods, but irrigation water allocation for wheat is likely to increase by 17.0–45.3 % in future periods.

## Background

Global food security threatened by climate change is one of the serious challenges in the twenty-first century to supply sufficient food for the burgeoning population while sustaining the already stressed environment. Changes in temperature and precipitation due to global climate change may have serious impacts on hydrologic processes, water resources availability, irrigation water demand, and thereby affecting the agricultural production and productivity. Meanwhile, climate variability is one of the most significant factors influencing year to year crop production, even in high yielding and high-technology agricultural areas (Kang et al. 2009). There are reports suggesting that decline in grain yields of rice and wheat in Indo-Gangetic Plains (IGP) could have been partly due to weather changes (Aggarwal et al. 2004).

Agricultural productivity is sensitive to climate change due to direct effects of changes in temperature, precipitation and carbon dioxide concentrations, and also due to indirect effects through changes in soil moisture and the distribution and frequency of infestation by pests and diseases (Mendelsohn 2014). The increase in temperature under climate change scenarios is expected to increase the evapotranspiration (ET) demand. Therefore, understanding the impacts of climate change on crop production and water resources is of utmost importance for developing possible adaptation strategies.

Various studies conducted to study the effects of climate change on the crop production showed that the effect of climate change on crop production varied with the climate change scenario used, current climate, cropping systems, management practices and also from region to region (e.g., Islam et al. 2012, 2014; Hillel and Rosenzweig 2011; Ko et al. 2011; Rosenzweig and Parry 2004). Naresh Kumar et al. (2013) reported decrease in irrigated rice yields in India by about 4, 7, and 10 % during the 2020s (2010–2039), 2050s (2040–2069), and 2080s (2070–2099), respectively. Rainfed rice yields in India were projected to decrease by about 6 % during the 2020s scenario, but during the 2050s and 2080s decrease was projected to be marginal (<2.5 %). Naresh Kumar et al. (2014) reported 6–23 and 15–25 % reduction in the wheat yield in India during 2050s and 2080s, respectively, under projected climate change scenarios. Mishra et al. (2013) reported a change in the rice yield in the range of −4.7 [lower Indo-Gangetic Basin (IGB)] to −23.8 (upper IGB) and 1.2 (lower IGB) to −5.9 % (upper IGB) under the REMO and HadRM3 projected climate change scenarios, respectively. They also reported a change in wheat yield in the range of −1.7 (lower IGB) to −12.9 (upper IGB) and 5.4 (lower IGB) to −6.1 % (upper IGB) under REMO and HadRM3 projected scenarios, respectively. These results indicate need for region specific studies for developing proper adaptation strategies.

Soil and Water Assessment Tool (SWAT) is a comprehensive, hydrological model that incorporates hydrological, chemical, and ecological processes and management practices in watershed simulation and analysis (Arnold et al. 1998; Neitsch et al. 2011). It simulates the plant growth by simplifying the generic crop growth module from the erosion productivity impact calculator (EPIC) model (Neitsch et al. 2011). This model has been successfully applied for studying impact of climate change on water resources (e.g., Gosain et al. 2011; Singh and Gosain 2011; Narsimlu et al. 2013) as well as in crop production (e.g., Lakshmanan et al. 2011; Bhuvaneswari et al. 2013) in different river basins of India. Lakshmanan et al. (2011) modelled the hydrology and rice yield of the Bhavani basin in Tamil Nadu, India and showed that the SWAT can be employed under different climate change as well as management scenarios for developing adaptation strategies to sustain rice production under changing climate scenarios. SWAT has also been used to assess the impact of El Niño/Southern Oscillation on hydrology and rice productivity in the Cauvery basin in India (Bhuvaneswari et al. 2013). The main objective of this paper is to assess the climate change impact on rice and wheat yield in the Gomti River basin using the SWAT hydrological model and climate change projections from Model of Interdisciplinary Research on Climate (MIROC–HiRes) GCM.

## Methods

### Description of the study area

**Fig. 1:**
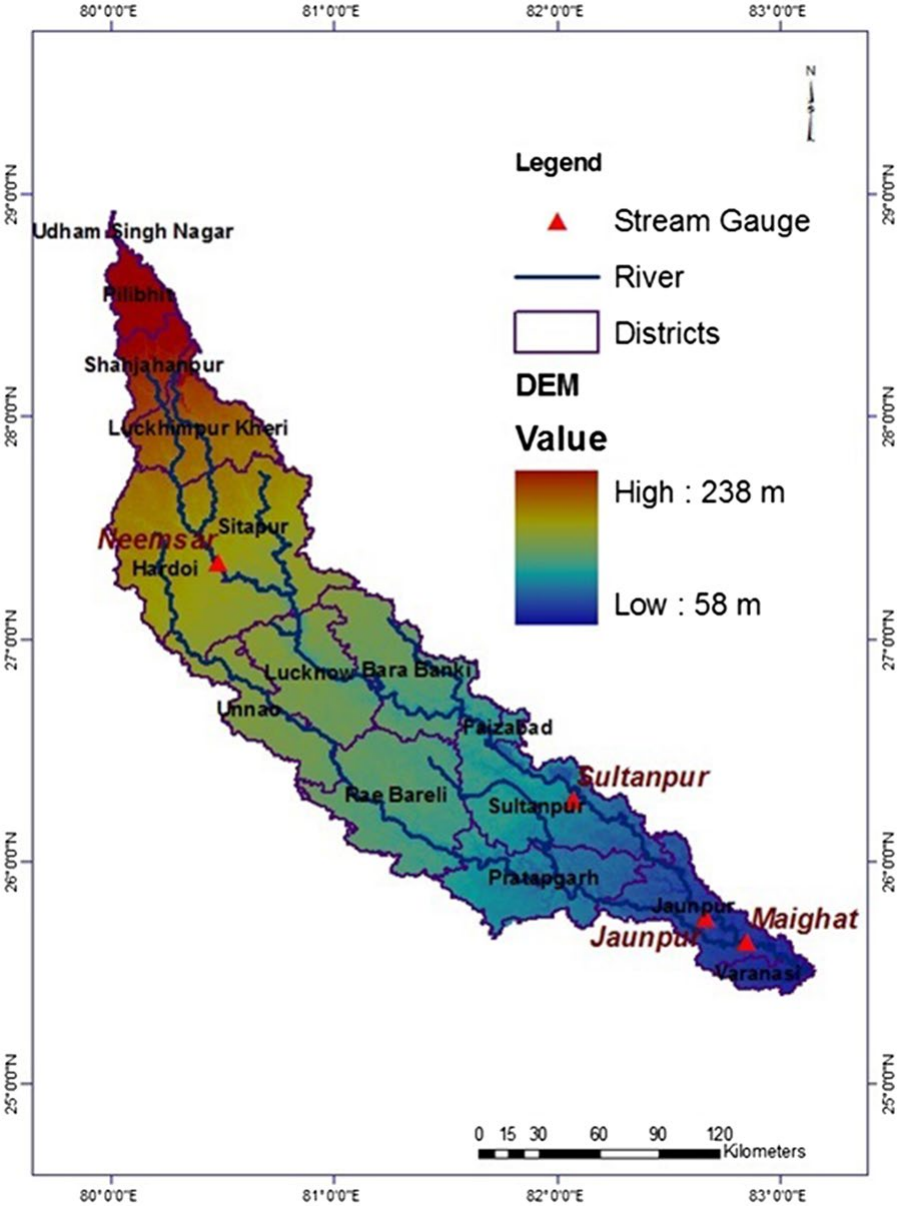
Location map of Gomti River basin with distribution of gauging stations and major stream network, and district boundaries in the basin

The Gomti River basin lies mainly in Uttar Pradesh (UP) and is spread over an area of 30,437 km^2^ (Dutta et al. 2011). Topography of the basin is undulating, and the elevation ranges from 58 to 238 m above mean sea level (MSL) (Fig. 1). The climate of the basin is semi-arid to sub-humid tropical with average annual rainfall varying from 850 to 1100 mm. This river is one of the important tributaries of the Ganga River and it meets the main Ganga River at Kaithi in Varanasi (UP) after flowing 960 km in south, south-east direction (Abeysingha et al. 2015).

### SWAT model description

The SWAT model was developed for exploring the effects of climate and land management practices on water, sediment and agricultural chemical yields (Arnold et al. 1998). This is a watershed scale model and simulates the hydrological cycle, plant growth cycles, transportation of sediment, and agricultural chemical yields on a daily time step. In SWAT, the watershed is divided into a number of sub-watersheds that are further subdivided into hydrologic response units (HRUs) based on unique soil, slope and land-use characteristics. The model simulates hydrology at each HRUs using the water balance equation. In present study, we used the latest version of SWAT (SWAT-2012.10_1.14) with ArcGIS (ver.10.1) interface.

The SWAT model provides different methods to estimate surface runoff, evapotranspiration and channel routing (Neitsch et al. 2011). We used the SCS curve number procedure (USDA SCS 1972), the Penman–Monteith method (Monteith 1965), and variable storage coefficient method (William 1969) for the estimation of runoff, evapotranspiration and channel routing, respectively. Actual evapotranspiration was estimated based on methodology developed by Ritchie (1972).

In the SWAT model, crop growth is computed based on EPIC crop growth model. The crop growth model initially computes the potential crop growth under ideal growing conditions by simulating leaf area development, light interception, and conversion of intercepted light into biomass assuming a species-specific radiation-use efficiency (RUE) (Neitsch et al. 2011). If there are constraints imposed by water, temperature, and nutrients in a simulation day, the SWAT model simulates actual crop growth with the applicable stress factors. SWAT considers the temperature stress as a function of the daily average air temperature and the optimal temperature for plant growth. As the air temperature diverges from the optimal, plant begins to experience stress (Neitsch et al. 2011). Following equations are used to determine the temperature stresses (Neitsch et al. 2011):

1 $${\text{tstrs}} = 1\quad {\text{when}}\;\; \bar{T}_{av} \le T_{base}$$

2 $${\text{tstrs}} = 1 - { \exp }\left\lfloor {\frac{{ - 0.1054 \cdot (T_{opt} - \bar{T}_{avg} )^{2} }}{{(T_{opt} - \bar{T}_{avg} )^{2} }}} \right\rfloor \quad {\text{when}}\;\; T_{base} < \bar{T}_{av} \le T_{opt}$$

3 $${\text{tstrs}} = 1 - { \exp }\left\lfloor {\frac{{ - 0.1054 \cdot (T_{opt} - \bar{T}_{avg} )^{2} }}{{(2 \cdot T_{opt} - \bar{T}_{avg} - T_{base} )^{2} }}} \right\rfloor \quad {\text{when}}\;\; T_{opt} < \bar{T}_{av} \le 2T_{opt} - T_{base}$$

4 $${\text{tstrs}} = 1\quad {\text{when}}\;\;\bar{T}_{av} > 2T_{opt} - T_{base} .$$

where, tstrs is the temperature stress for a given day expressed as fraction of optimal plant growth, $\bar{T}_{av}$ is the mean air temperature for a day (°C), *T*_*base*_ is the plant’s base or minimum temperature for growth (°C), and *T*_*opt*_ is the plant’s optimal temperature for growth (°C).

### SWAT model set-up

**Fig. 2:**
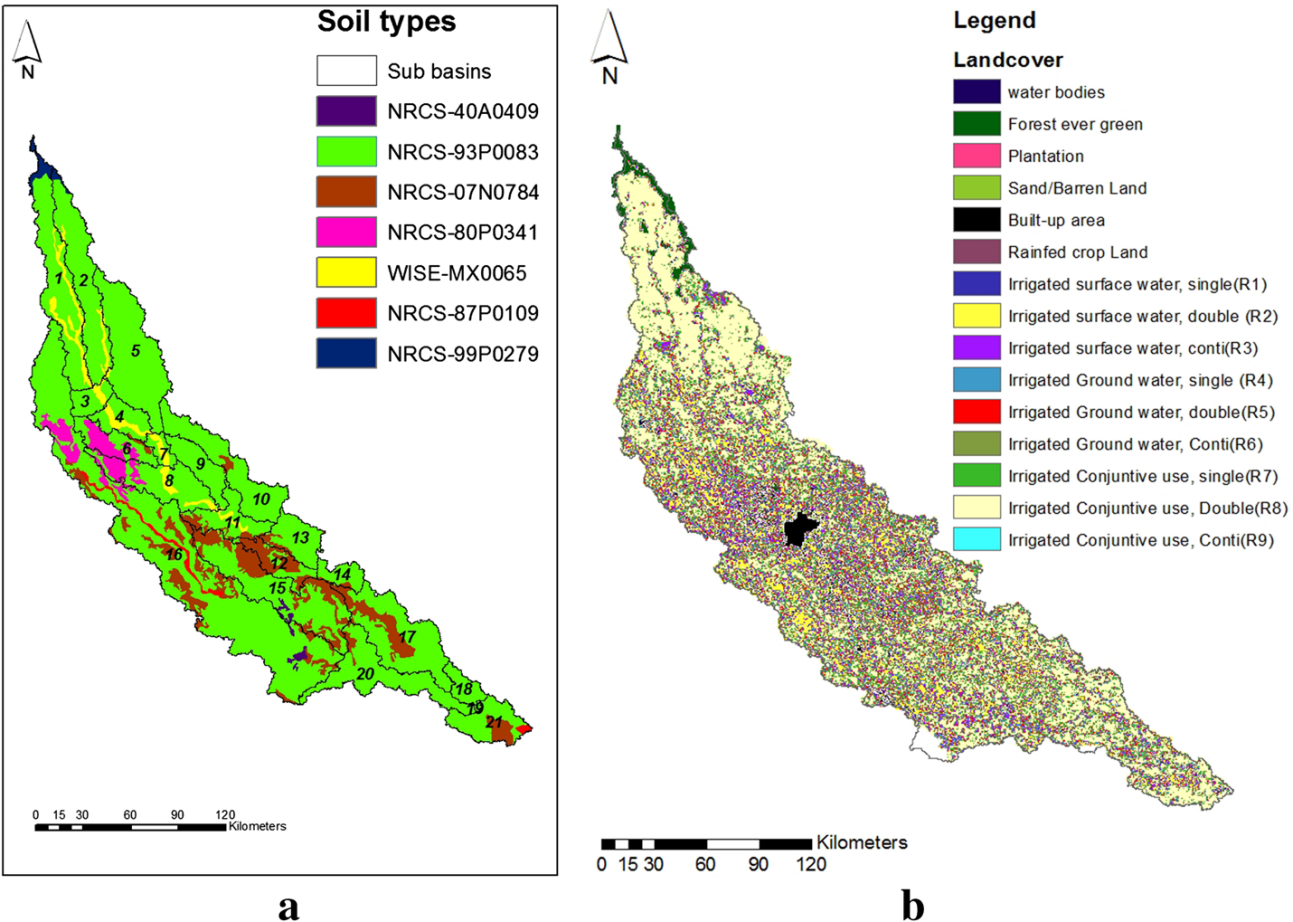
Soil and landuse map of the Gomti River basin (1–21 denotes the sub basin numbers). **a** Soil types and sub basin map. **b** Land use land cover map

For SWAT simulation, the basin, sub basins and stream network were delineated from the 90 m × 90 m shuttle radar topography mission (SRTM) (Jarvis et al. 2008) digital elevation model (DEM) (http://gisdata.usgs.gov/website/Hydro-SHEDS/), Gomti River basin was divided into 21 sub-basins (Fig. 2a) at the SWAT watershed delineation process and sub-basin discretization for spatial aggregation, and was further divided into 296 HRUs at HRU definition and analysis. The soil types of the study area were extracted from a soil map (78 × 78 m resolution) of the Ganga River basin (http://gisserver.civil.iitd.ac.in/grbmp/iitd.htm) which has been digitized from the soil map of National Bureau of Soil Survey and Land Use Planning (NBSS&LUP) (Fig. 2a). Soils of the area were predominantly alluvial, deep soil. Soil properties were also taken from the same NBSS&LUP soil map. For land use and land cover data, the satellite remote sensing derived International Water Management Institute (IWMI) land-cover map (Thenkabail et al. 2009) of the study area at 56 × 56 m resolution was used. The predominant land use in the basin was agriculture, with 59.4 % of the area occupying irrigated conjunctive use double cropping (SWAT model class, R-08), and 32.7 % area occupying irrigated surface water, double cropping (R-02) (Fig. 2b). Other land use categories were forests (2.6 %), irrigated surface water continuous crop lands (1.3 %—R03), barren lands (1.1 %) etc. Climate data required by the model are daily precipitation, maximum and minimum air temperature, solar radiation, wind speed, and relative humidity. Historical daily precipitation and air temperatures of 14 districts (Barabankki, Hardoi, Kheri, Lucknow, Pilibhit, Shahjahanpur, Sitapur, Unnao, Udham Singh Nagar, Faizabad, Pratapgarh, Rae Bareli, Sultanpur, and Jaunpur) covering the entire basin for the period 1982–2010 were obtained from the National Initiative on Climate Resilient Agriculture web portal (NICRA, http://www.nicra-icar.in/nicrarevised/). Daily values of solar radiation, wind speed, and relative humidity were generated using long term statistics through the WXGEN weather generator (Sharpley and Williams 1990) in SWAT.

### Inputs for simulation of crops in the basin

We assigned crops to IWMI land use and land cover map of the Gomti River basin considering the cropping pattern of Uttar Pradesh (UP) assessed using IRS-P6 (AWiFS) data (Singh et al. 2011). They showed that the rice–wheat is the main cropping pattern and the order of the cropping pattern in UP in terms of area is rice–wheat > sugarcane > rice–pulses > sugarcane–wheat etc. The land cover category R-08 was assigned to irrigated rice (*kharif*) and wheat (*rabi*). The R-02 category was assigned to rice (*kharif*) and pulses (*rabi*) and R-03 category was assigned to sugarcane (*annual*) crop. R-08, R-02, and R-03 occupy 59.58, 32.45, and 1.38 % areas, respectively, in the basin. The management inputs on planting, harvesting and irrigation were obtained from the available literature pertaining to the area (Hobbs et al. 1992; Gangwar and Singh 2011). Considerable part of the Gomti River basin is supplied with canal water from Sharda Sahayak canal system. Therefore, water source for the simulation of the rice, sugarcane, and pulse (lentil) was considered as canal water in HRUs, where canal is located, and SWAT recognized the source as outside unlimited source. For the other rice growing HRUs where canal is not located, source of irrigation was considered as shallow aquifer located in the same sub-basin. For irrigation of wheat, auto irrigation option of SWAT was used in which source of irrigation water was considered as shallow aquifer located in the same sub basin. For auto-irrigation, the plant water stress threshold that triggers irrigation was set to 0.9 initially (Arnold et al. 2012). The HRUs under sugarcane and pulse (lentil) crops were irrigated similarly as that of area under wheat. The sugarcane and pulse crops were simulated as part of land use category in the model setup but they are not calibrated and validated and hence not reported in this paper. We selected automatic fertilization option for fertilizing the crops because of the difficulty in obtaining fertilizer schedules for each crop at each HRUs.

Paddy fields in the SWAT model are treated as a pothole, like an impounded or depression area. Impound operation was given before planting, and release operation was given 5 days before harvesting of paddy for each rice growing HRUs. Maximum volume of water stored in the pothole was fixed to 60 mm and the fraction of area that drains to pothole was initially set as 0.8. Moreover, at the initial stage of the modelling, initial leaf area index (LAI) and biomass of rice were set as 1.1 and 800 kg/ha, respectively as rice is mostly a transplanted crop (Kaur et al. 2003). However, these values were lowered to 0.9–1 and 700–780 kg/ha, respectively while calibrating the model to match the observed and simulated rice yield of different districts.

### Climate change scenarios

Future climate projections of the “Model of Interdisciplinary Research on Climate (MIROC)” GCM, from the *World Climate Research Programme’s (WCRP’s)* Coupled Model Intercomparison Project Phase 3 (CMIP3) climate projections *multi*-*model dataset* (Meehl et al. 2007), were used to develop climate change scenarios. The MIROC3.2 model, developed at the National Institute for Environmental Studies of Japan, has been found to perform quite satisfactorily, with larger pattern correlation and smaller root-mean-square differences for India (Anandhi and Nanjundiah 2014; Das et al. 2012). Using the MIROC 3.2 (HiRes) monthly projection for three Special Report on Emission Scenarios (SRES) emission scenarios, namely A2 (high), A1B (medium) and B1 (low), daily rainfall and temperature time series were generated for three future periods of 2020s (2010–2039), 2050s (2040–2069), and 2080s (2070–2099). We used the most commonly used perturbation (or delta change) method (Hay et al. 2000; Ragab and Prudhomme 2002; Khoi and Suetsugi 2012) for generating the climate change scenarios. These perturbed rainfall and temperature data of 12 stations, spread over the basin, were input to the calibrated and validated SWAT model and model was run for the each emission scenarios and time periods separately. The results were analysed separately for each of the emission scenarios and time periods. The results in each case were expressed as cumulative distribution functions (CDFs) and as percentage change with respect to the baseline period.

### Model calibration and validation for streamflow

The SWAT model was calibrated and validated for the streamflow at four spatially distributed gauging stations, Neemsar, Sultanpur, Jaunpur and Maighat (Fig. 1) based on the monthly streamflow data obtained from Central Water Commission (CWC), Ministry of Water Resources, River Development and Ganga Rejuvenation, Government of India. Eight years warm-up period (1982–1990) was considered prior to the model simulation starting date to stabilize the model initial condition. Before calibration and validation, a sensitivity analysis was performed using SUFI2 in SWAT CUP auto calibration tool (version 5.1.7) (Abbaspour 2012). Most sensitive hydrological parameters were then adjusted manually considering their limits (Moriasi et al. 2007) and taking the support from SWAT CHECK which is embedded in the SWAT 2012. After calibrating the crop parameters manually (as discussed in section “Rice and wheat yield calibration and validation”) final adjustments for the hydrological parameters were done by matching observed and simulated streamflow data of four different gauging stations. The model was calibrated using the data for 1990–2000 for all the four gauging stations. The model validation was done using 2001–2008 data for all the gauging stations, except for Maighat station where data for the period 2001–2006 was used for model validation. The Nash–Sutcliffe efficiency (NSE), coefficient of determination (*R*^2^), RMSE-observations standard deviation ratio (RSR), and percent bias (PBIAS) were used as benchmarking indices to assess the goodness of fit of simulated and observed streamflow.

### Rice and wheat yield calibration and validation

Annual yields of both rice and wheat were calibrated by manually adjusting the parameters such as harvest index (HVSTI), biomass energy ratio (BIO_E), Auto_NSTRS (nitrogen stress factor that triggers fertilization), Auto_WSTRS (water stress threshold that trigger irrigation), for both the crops. In addition to the above parameters, the planting and harvesting dates and heat unit to maturity for wheat and initial LAI and biomass for rice were also considered for calibrating the model.

The model was calibrated for rice using the yield data for the period 1995–2002 and validated using the data for the period 2003–2008. Similarly, calibration of the model for wheat was done using the yield data for 1996, 1998–2003, and data for 2004–2009 periods were used for model validation. Reported rice and wheat yield data of four districts (Lucknow, Sultanpur, Barabanki, and Jaunpur) in the basin, collected from the State Department of Agriculture, Lucknow, Uttar Pradesh, were used for calibration and validation of the model. Area weighted average simulated yield of each HRUs in relevant districts were compared with the reported district average yield for calibration and validation of SWAT model. The differences between the measured and simulated yields were evaluated by using *t* test statistics (*p* > 0.05) and PBIAS (<10 %). These statistics for SWAT crop yield calibration has been reported in previous studies too (Hu et al. 2007; Nair 2010; Ahmad et al. 2011).

## Results and discussion

### Streamflow calibration and validation

Streamflow at the four gauging stations were calibrated and validated for the most sensitive parameters by matching measured and simulated streamflow of the four gauging stations located in the basin. The most sensitive factors considered for calibration and validation are: Base flow alpha factor (ALPHA_BF), available water capacity (SOL_AWC), plant uptake compensation factor (EPCO), delay of time for aquifer recharge (GW_DELAY), aquifer percolation coefficient (RCHRG_Dp), groundwater ‘revap’ coefficient (GW_REVAP), soil evaporation compensation factor (ESCO), curve number (CN2), threshold water depth in the shallow aquifer for flow (GWQMIN), threshold water level in shallow aquifer for revap (REVAPMIN), saturated hydraulic conductivity (SOL_K), Manning’s “n” value for overland flow (OV_N), maximum canopy storage (CANMAX), channel effective hydraulic conductivity (CH_K2), bulk density (SOL_BD). During calibration period, the performance indicators *R*^2^, NSE, RSR, and PBIAS values were in the range of 0.66–0.78, 0.62–0.74, 0.51–0.61, and −0.25 to 14, respectively. During validation period, the same indicators values ranged from 0.57 to 0.73, 0.51 to 0.72, 0.50 to 0.68, and −1.1 to 17.7, respectively (Table 1).

**Table 1:** Model calibration and validation performance statistics for monthly streamflows at the four gauging stations

	Calibration				Validation			
	NSE	RSR	PBIAS (%)	*R* ^2^	NSE	RSR	PBIAS (%)	*R* ^2^
Neemsar	0.72	0.52	−0.25	0.73	0.72	0.53	−1.1	0.76
Sultanpur	0.74	0.51	14.0	0.78	0.64	0.50	3.2	0.69
Jaunpur	0.62	0.61	12.4	0.66	0.54	0.67	4.0	0.63
Maighat	0.72	0.53	9.76	0.77	0.51	0.68	17.7	0.57

According to the performance rating suggested by Moriasi et al. (2007), model performance was good (0.65 < NSE ≤ 0.75; 0.5 < RSR ≤ 0.6; and ±10 < |PBIAS| ≤ ±15) during both calibration and validation phase at Neemsar and Sultanpur, and during calibration phase at Maighat. The model performance was found to be satisfactory (0.5 < NSE ≤ 0.65; 0.6 < RSR ≤ 0.7; and ±15 < |PBIAS| ≤ ±25) during the both calibration and during validation phase at Jaunpur, and during validation phase at Maighat gauging station. The statistical indicators of the Maighat gauging station, which is located at the downstream end of the basin and reflecting the outflow from the entire basin, indicates that model performance is good at the calibration phase but satisfactory at the validation phase. Based on threshold *R*^2^ or E (model efficiency) value, Parajuli (2010) categorized model performance for monthly streamflow as excellent (≥0.90), very good (0.75–0.89), good (0.50–0.74), fair (0.25–0.49), poor (0–0.24), and unsatisfactory (<0). According to these criteria, SWAT model performed reasonably well in simulating the streamflow for the entire Gomti basin with R^2^ and NSE values ≥0.50 for all the sub-basins both during calibration and validation. Overall, the SWAT model exhibits satisfactory performance in simulating monthly streamflows for the entire Gomti River basin as this study used observed streamflow of four spatially distributed gauging stations and long term records which cover both dry and wet years.

### Rice and wheat calibration and validation

Simulated rice and wheat yield were compared with the reported yields of different districts located within the basin. According to *t* test statistics (*p* > 0.05) and PBIAS (<10 %) evaluation statistics, SWAT model performed reasonably well in all the four districts in simulating the rice yield during calibration period (Table 2). Further, model performed reasonably well during validation phase except at Jaunpur district where PBIAS > 10 (Table 2) and Lucknow where *t* test value is <0.05. Similarly, mean wheat yield was also simulated reasonably well by the model in both calibration and validation phases as far as *t* test values are concerned. However, PBIAS was slightly higher than 10 at calibration phase in Lucknow, and during validation phase in Lucknow, Barabanki and Sultanpur (Table 2). The over estimation (rice: 0.005–0.85 t/ha and wheat: 0.04–1.5 t/ha) and underestimation (rice: 0.01–0.48 t/ha and wheat: 0.065–1.74 t/ha) of rice and wheat yield during some years of calibration and validation periods could be attributed to different management practices followed e.g., tillage operations, crop rotations, depth and frequency of water application and planting dates etc. in different districts. However, SWAT could simulate the long term average yield quite well as shown in the mean statistics (Table 2). Since long term (30 years) mean yield for future as well as baseline period were used for climate change impact studies, the performance of the SWAT model in simulating mean yield may be considered as quite satisfactory.

**Table 2:** Rice and wheat calibration and validation performance statistics

Districts	Calibration				Validation			
	Reported mean yield (t/ha)	Simulated mean yield (t/ha)	t-statistics	PBIAS	Reported mean yield (t/ha)	Simulated mean yield (t/ha)	t-statistics	PBIAS
*Rice*								
Lucknow	1.68	1.78	0.29	−5.8	1.96	1.77	0.03*	10.0
Barabanki	2.01	1.98	0.66	1.6	2.18	1.98	0.12	9.0
Sultanpur	2.05	2.12	0.52	−3.1	2.14	2.18	0.37	−6.5
Jaunpur	2.13	2.13	0.99	0.01	1.88	2.13	0.16	−13.7
*Wheat*								
Lucknow	2.35	2.74	0.18	−14.7	2.51	2.83	0.10	−10.5
Barabanki	2.59	2.37	0.47	8.5	2.93	2.45	0.16	16.4
Sultanpur	2.51	2.32	0.52	7.3	2.70	2.30	0.15	14.7
Jaunpur	2.40	2.41	0.72	3.9	2.50	2.40	0.72	3.9

* Indicates *t* values significant at *p* < 0.05

### Temperature and rainfall change during the rice and wheat growing seasons

**Fig. 3:**
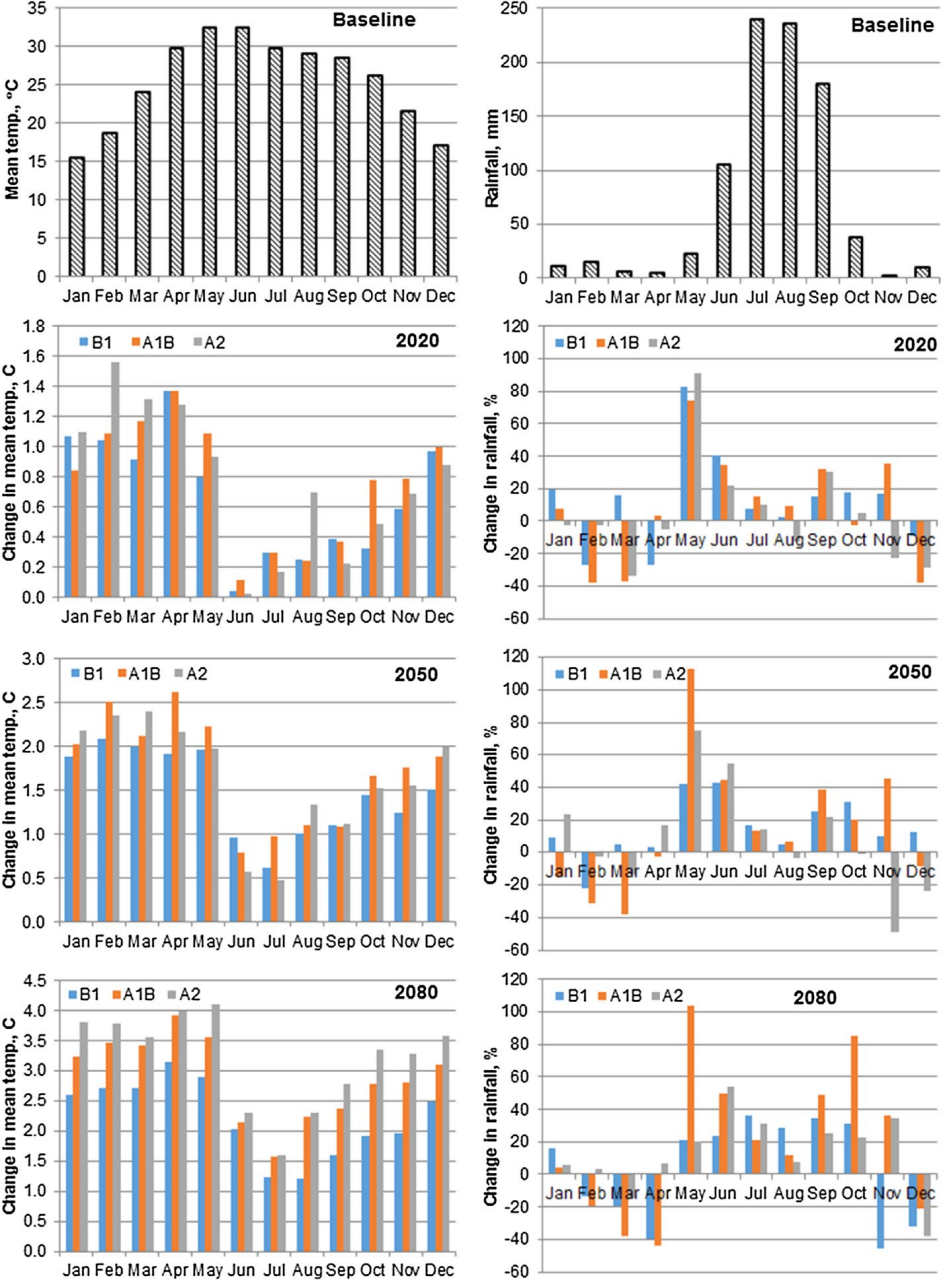
Projected changes in mean temperature and rainfall in the basin

Mean temperature changes in the basin varied from 0.02 to 1.56, 0.47–2.60, and 1.21–4.10 °C during 2020s, 2050s, and 2080s, respectively under different emission scenarios (Fig. 3). During the rice growing months (June–October), the changes in mean temperature varied from 0.02 to 0.77, 0.46 to 1.66, and 1.21 to 3.34 °C during 2020s, 2050s, and 2080s, respectively, depending upon the different emission scenarios. As shown in Fig. 3, the mean temperature changes during wheat growing season (November–April) were higher than the projected temperature change for rice growing season. It varied from 0.58 to 1.56, 1.23 to 2.60 and 1.96 to 3.98 °C, during 2020s, 2050s, and 2080s, respectively.

As shown in Fig. 3, there is increase in rainfall in the basin during most of the months. The changes in the mean monthly rainfall in the basin varied in the range of −38.1 (Dec) to 91.5 (May), −48.8 (Nov) to 112.7 (May), and −45.8 (Nov) to 103.6 % (May) during 2020s, 2050s, and 2080, respectively, depending upon the different emission scenarios. During the rice growing period (June–October), there is increase in rainfall under all three emission scenarios for all the three future periods in most of the months, except for the month of August in which there is decrease in rainfall under the A2 emission scenario during 2020s and 2050s (Fig. 3). This decrease in rainfall during August is 13 and 4 % during 2020s and 2050s, respectively. Mean rainfall changes during the wheat growing months are different during different month and also under different emission scenarios (Fig. 3). Overall, month of December, February, March and April showed a decrease in mean rainfall during all the three future period for most of the emission scenarios. In the month of January, rainfall is likely to increase during 2080s for all emission scenarios in the entire basin. This decrease in rainfall during wheat growing season is likely to impact the yield as well as irrigation water demand.

### Impact of rainfall and temperature change on rice and wheat production

#### Rice

**Fig. 4:**
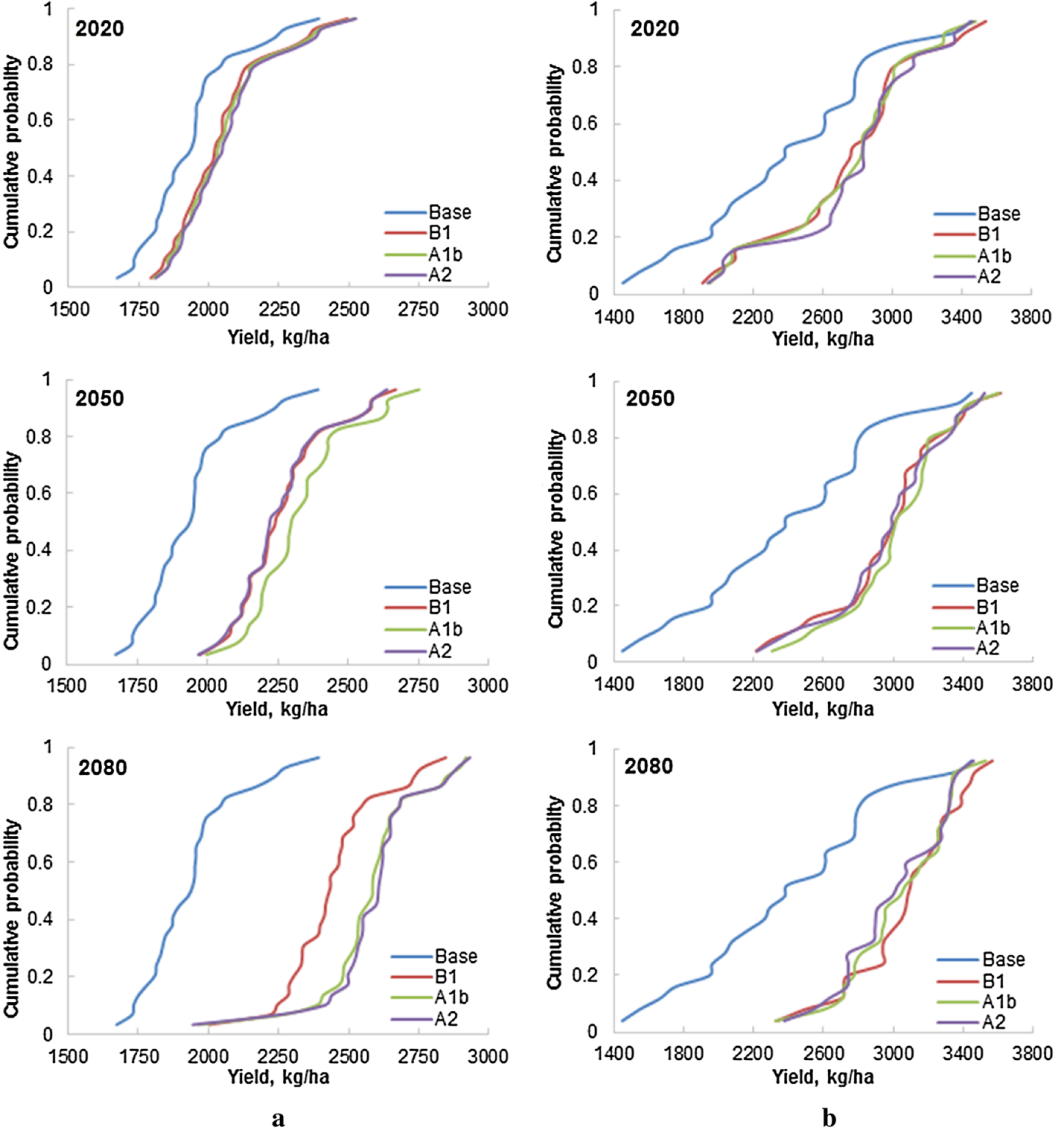
Cumulative distribution function of mean rice and wheat yield in response to climate change scenarios. **a** Rice. **b** Wheat

**Fig. 5:**
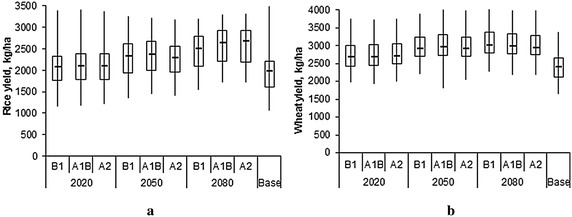
Rice and wheat yield variations in different HRUs within the basin under changing climate scenarios. **a** Rice. **b** Wheat

**Fig. 6:**
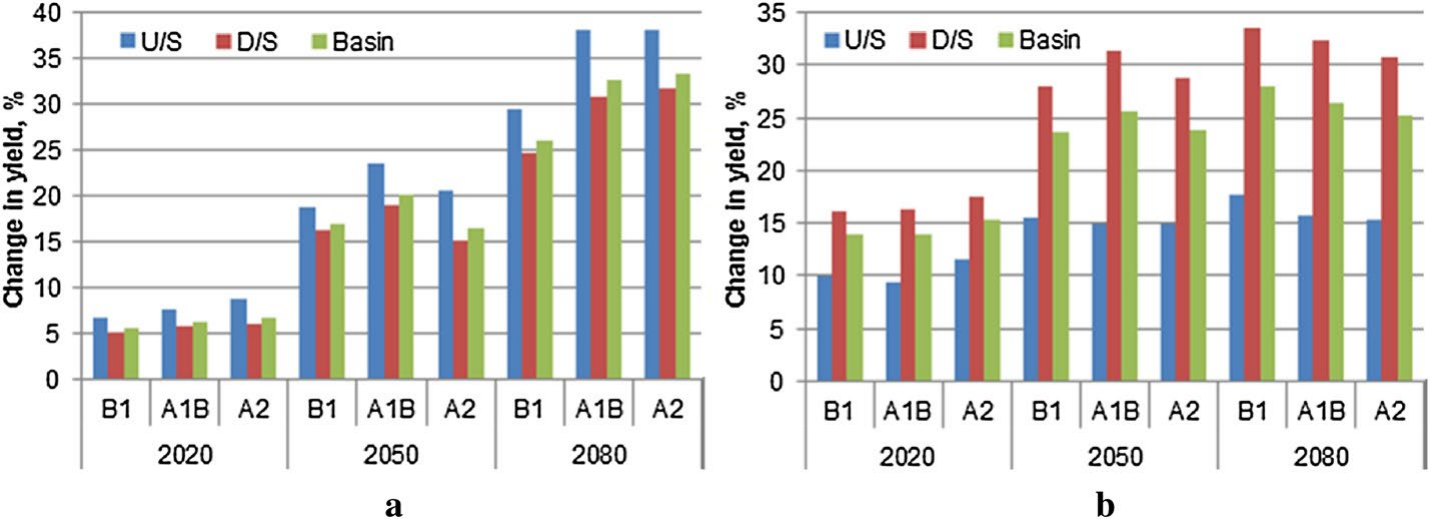
Changes in rice and wheat yield under different climate change scenarios. **a** Rice. **b** Wheat

The cumulative distribution function plotted using the 30 years simulated rice yield data, clearly indicates an increase in rice yield under the changing climate scenarios for the all the three future periods (2020s, 2050s, and 2080s), with comparatively greater increase during 2080s as compared to 2050s and 2020s (Fig. 4a). During 2020s, there is marginal increase in yield and the increase in yield remained almost same for all the three emission scenarios. Further, analysis of HRU wise simulation results indicated large variations (1162.5–3401.5 kg/ha) in the simulated yields amongst different HRUs (Fig. 5a) under different climate change scenarios. The median yield in the basin under different climate change scenarios ranged from 2077.8 (B1) to 2086.2 kg/ha (A2), 2286.6 (A2) to 2363.5 kg/ha (A1B), and 2503.5 (B1) to 2567.2 (kg/ha) (A2) during 2020s, 2050s, and 2080s, respectively, as compared baseline median yield of 1965.4 kg/ha. The large variation yield in different HRUs within basin is mainly due to different soil characteristics, input use and management practices etc. Overall, change in the mean rice yield in the basin varied from 5.5 to 6.7, 16.6 to 20.2 and 26.0 to 33.4 %, during 2020s, 2050s and 2080s, respectively (Fig. 6a). It is also to be noted that increase in yield is slightly higher at the upstream basin as compared to the downstream basin. At the upstream basin the increase in rice yield ranged from 6.6 to 8.7, 18.8 to 23.5 and 29.4 to 38.1 % during 2020s, 2050s and 2080s, respectively, whereas at the downstream basin the increase in rice yield ranged from 5.1 to 6.0, 15.1 to 19.0 and 24.7 to 31.7 % during 2020s, 2050s and 2080s, respectively. Relatively higher increase in yield at the upstream basin may be attributed to the higher rainfall at the upstream basin (Figs. 3, 8). As we assumed that Sardha Sahayak canal supplies irrigation water without any limitation even in future time and rainfall is likely to be substantially increasing during future time periods over the rice growing season (Fig. 3), water stresses may not limit the paddy growth and development. The higher increase in rice yield towards the end of the century may be attributed to higher increase of rainfall during the end of the century (Fig. 6a).

Matthews et al. (1995) reported a reduction in rice yield of about 5 % per degree rise in mean temperature above 32 °C. Krishnan et al. (2007) predicted average rice yield changes of −7.20 and −6.66 % with every 1 °C increase in temperature at the current level (380 ppm) of CO_2_ using the ORYZA1 and INFOCROP rice models, respectively, in the eastern region of India. They also reported a maximum gain of 11.08 % at Jorhat, where the climate is warm moist perhumid type had the maximum temperature of about 28 °C and a minimum temperature of 19 °C only as the temperature at the time of flowering affects the spikelet fertility and hence the yield. In our study basin, number of days with *T*_*max*_ > 30 °C remained almost same as that of baseline scenarios (Table 3). Analysis of the mean number of days with maximum temperature (*T*_*max*_) greater than 30 °C during the rice growing season indicated that that the number of days with *T*_*max*_ > 30 °C ranged from 95 to 99 days in the basin during future periods as against baseline of 93 days (Table 3). Temperature changes during the rice growing period were not significantly high in the basin (Fig. 3). The optimum temperature for the normal development of rice ranges from 27 to 32 °C (Yin et al. 1996; Shah et al. 2011). The projected mean temperatures in the basin during July–Oct ranged from 26.5 to 30, 27.6 to 30.7 and 28.1 to 31.5 °C, respectively, which is within the optimum range (Yin et al. 1996; Shah et al. 2011) in most of the cases. Thus, temperature changes may not affect the growth and yield of paddy. As there is not considerable increase in number of days with *T*_*max*_ > 30 °C when compared to the baseline period, future maximum temperature may not affect the rice production considerably in the Gomti river basin. It has also been reported that changes in the minimum temperature is more crucial than a change in the maximum temperature for rice with decline in rice yield by 10 % for each 1 °C increase in the minimum temperature above 32 °C (Pathak et al. 2003). However, MIROC projected maximum values for minimum temperature during the rice sensitive growing months were 26.04, 26.94 and 28.43 °C, for 2020s, 2050s and 2080s, respectively. Therefore, rice yield may not be negatively affected with these projected changes of temperature. Further, Saseendrain et al. (2000) reported an exponential increase in rice yield due to increase in rainfall above the observed values. Increase in temperature mostly remaining within the optimum limit as compared to the baseline together with the projected increase in rainfall under all three emission scenarios for all the three future periods in the basin (Fig. 3) probably contributed to the gain in the rice yield. In SWAT all stresses including water stress integrates together and influence on the growth and yield. Therefore, the positive effects of water in rice growth and development might outweigh the stress due to higher temperature. Simulation studies conducted using different models and scenarios have projected decrease in rice yield in India as well as Indo-Gangetic basin too (Naresh Kumar et al. 2013; Mishra et al. 2013). The differences in results may be attributed to the different crop growth simulation models used as well as climate change scenarios used. For example, Mishra et al. (2013) based on DSSAT model simulation reported a greater decrease (4.7–23.8) when REMO projected climate change scenario were used. They reported a change in the rice yield in the range 1.2 (lower IGB) to −5.9 % (upper IGB) under the HadRM3 projected climate change scenarios, respectively.

**Table 3:** Average number of days with *T*_*max*_ > 30 °C, *T*_*max*_ > 18 °C during rice and wheat growing season respectively under different climate change scenarios

Scenarios	Time period	Number of days with *T*_*max*_ > 30 °C during rice growing season			Number of days with *T*_*max*_ > 18 °C during wheat growing season		
		Basin	Upstream	Downstream	Basin	Upstream	Downstream
Base	Base	93	91	94	150	148	152
A2	2020	95	94	96	152	151	153
	2050	97	97	98	153	153	154
	2080	99	99	99	154	154	155
A1b	2020	95	94	96	152	151	153
	2050	98	97	98	153	153	154
	2080	99	99	99	154	154	154
B1	2020	95	93	96	152	151	153
	2050	97	96	98	153	152	154
	2080	98	98	99	154	153	154

#### Wheat

Similar to rice, there is also increase in the wheat yield in the basin, with higher increase during 2080s as compared to 2020s (Fig. 4b). However, increase in yield under the different emission scenarios remained almost the same. Further, HRUs wise analysis showed large variation (1645–4026.9 kg/ha) in the yields of different HRUs depending upon the emission scenarios and future periods (Fig. 5b). The median yield of wheat in the basin varied from 2685.4 to 2704.2, 2899.0 to 2946.8 and 2940.9 to 3013.8 kg/ha during 2020s, 2050s and 2080s, respectively depending upon the different emission scenarios. The baseline period median yield was 2386.8 kg/ha. The increase in mean wheat yield in the basin varied in the range of 13.9–15.4, 23.6–25.6 and 25.2–27.9 % during 2020s, 2050s and 2080s. As opposed to rice, increase wheat yield was higher at the downstream basin as compared to the upstream basin (Fig. 6b). At downstream areas of the basin, increase in wheat yield varied from 16–17.6, 28.0–31.4 and 30.8–33.6 % during 2020s, 2050s and 2080s, respectively. Wheat yield at upstream increased from 9.4–11.5, 14.9–15.6 and 15.3–17.7 %, during 2020s, 2050s and 2080s, respectively. In this study, wheat was irrigated using the shallow aquifer water. The decrease in rainfall in the month of December, February and March–May not trigger a decrease in wheat yield as irrigation water is available from the shallow water aquifer. However, the wheat yield increase in the basin was not significant (9–20 %) and CV of wheat yield also varied from 19 to 32 %, whereas CV of rice varied only from 14 to 17 %.

Ortiz et al. (2008) reported that global warming may be beneficial for the wheat crop in some regions, but could reduce productivity in zones where optimal temperatures already exist. The projected absolute mean temperature during wheat growing stage was not significantly higher as compared to the model default *T*_*base*_ and *T*_*opt*_ for wheat, which are 0 and 18 °C, respectively. Similar to rice, for wheat growing season also increase in number of days with daily maximum temperature greater than wheat optimum temperature (i.e., 18 °C), were not considerable in any of the future scenarios in the basin. Number of days with *T*_*max*_ > 18 °C during wheat growing season ranged between 152 and 154 days during future periods as against 150 days during baseline period (Table 3). Temperatures greater than 34 °C have been found to decrease wheat yields up to 50 % due to increased leaf senescence (Asseng et al. 2011). However, in this study, MIROC temperature projection did not demonstrate greater temperature such as 34 °C even during 2080s for  wheat cropping season. Therefore, the projected increase in maximum temperature may not affect the growth and yield of wheat in the basin. In addition, simulated increase in yield gain might be due to increased rate of irrigation water application (as discussed below). Lv et al. (2013) reported increase in irrigated wheat yields in almost all regions China under full irrigation conditions. Similar to rice, Mishra et al. (2013) also reported increase in wheat yield using DSSAT model and HadRM3 projected climate change scenarios in lower Ganga basin. But, they reported contrasting results for upper Ganga basin, where wheat yield was projected to decrease with REMO based projections. Based on the simulations studies for northern India [Indo-Gangetic Plains (IGP)] to monitor rates of wheat senescence following exposure to temperatures greater than 34 °C with two commonly used process-based crop models (CERES-Wheat and APSIM), Lobell and Gourdji (2012) indicated that existing models underestimate the effects of heat on senescence. Porter and Gawith (1999) also suggested explicit consideration of extreme high temperature events to better understand the full response range of growth and development processes for wheat as extreme high temperature events have autonomous effects on grain production. As the simulated climate change impact varies across models due to differences in model structure and its parameterization (Asseng et al. 2013) and climate change scenarios used, multi-model ensemble analysis may be helpful for better quantification of climate change impact on crop yield and preparing adaptation plans.

### Change of irrigation and evapotranspiration

**Fig. 7:**
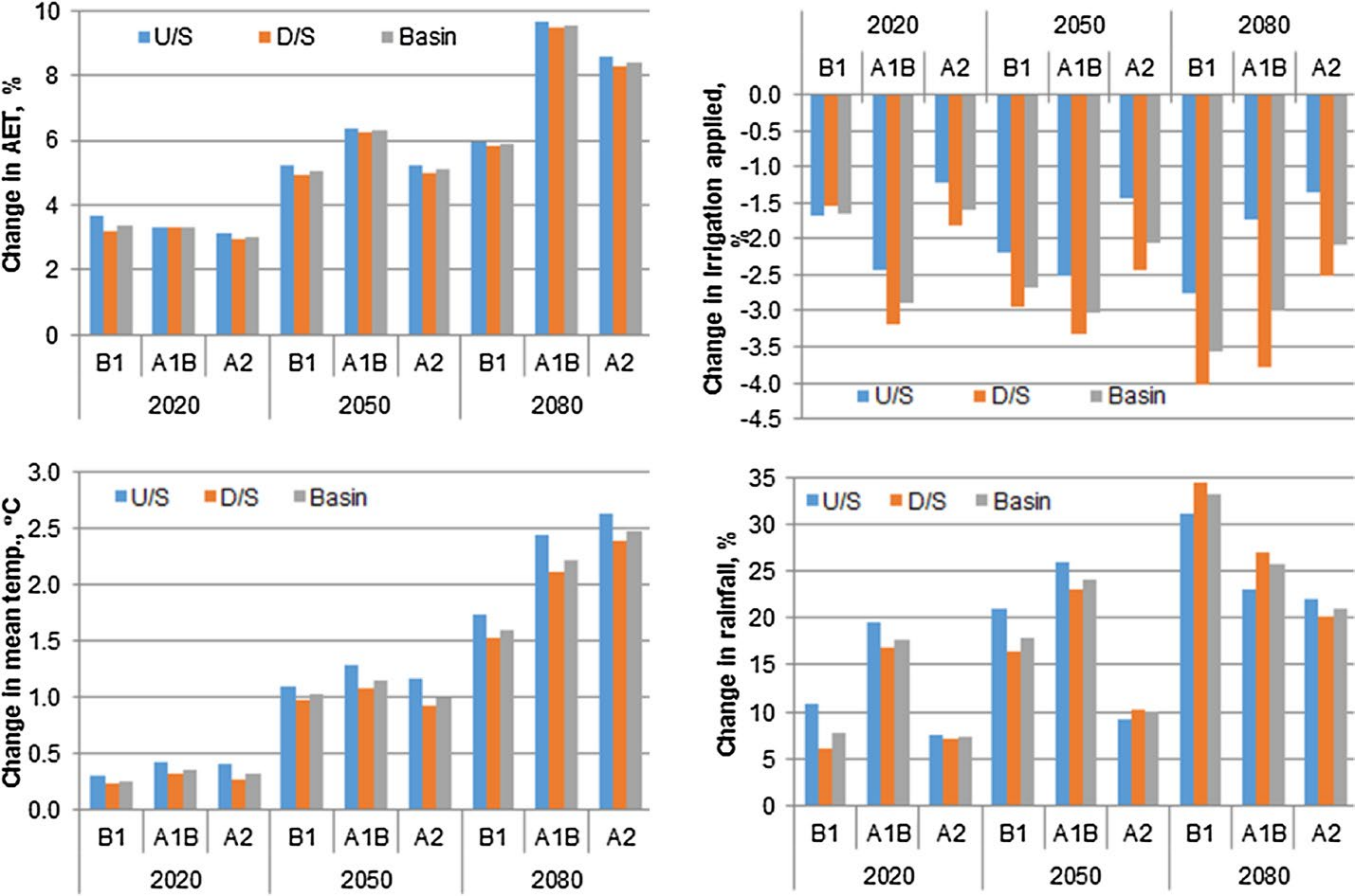
Change in AET, irrigation applied, seasonal temperature and rainfall change during rice growing season

**Fig. 8:**
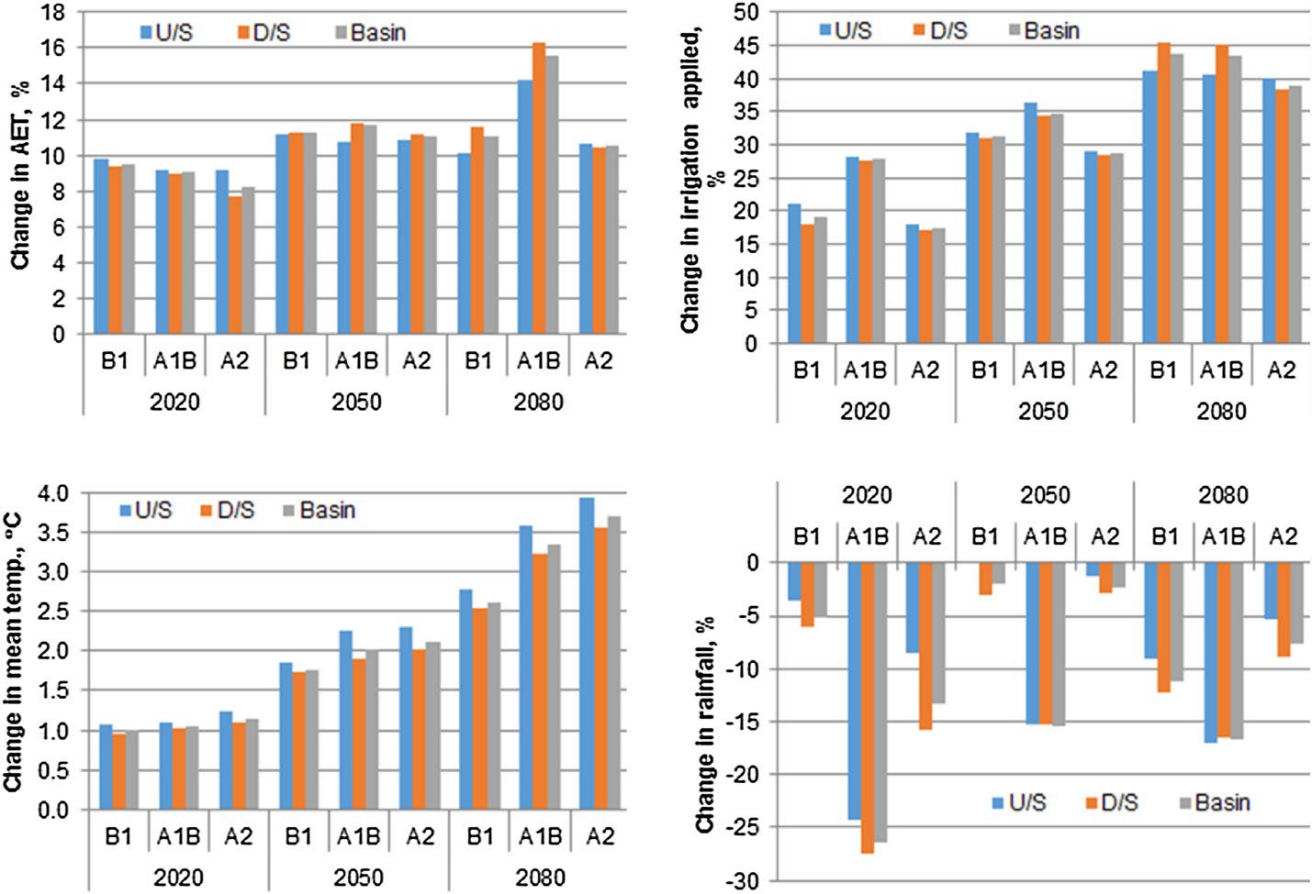
Change in AET, irrigation applied, seasonal temperature and rainfall change during wheat growing season

SWAT simulation showed increase in mean actual evapotranspiration for both wheat and rice as compared to the baseline (Figs. 7, 8). However, the increase in actual evapotranspiration for wheat is higher than that of rice. The increase in evapotranspiration during rice growing period in the basin varied in the range of 3–3.35, 5–6.3 and 5.9–9.6 % during 2020s, 2050s and 2080s, respectively, with slightly higher increase in the upstream basin as compared to the downstream basin (Fig. 7a).

Increased rainfall during rice growing period resulted in the decreased irrigation water application for rice. As per the SWAT simulation results, irrigation could be decreased slightly in future for the rice cultivation in this basin. There was approximately 1–4 % (<5 %) decrease in irrigation water application for rice during the future periods of 2020s and 2050s and 2080s. This decrease in irrigation water application is mainly due to increase in rainfall (Fig. 7a). The increase in rainfall in the basin during rice growing season varied in the range of 7.3–17.8, 9.9–24.0 and 20.9–33.2 % during 2020s, 2050s and 2080s, respectively. The increase in rainfall as well mean temperature is relatively higher at the upstream basins as compared to downstream basins during rice growing season.

As shown in the Fig. 8, the increase in actual evapotranspiration during wheat season varied from 7.8–9.8, 10.8–11.8 and 10.1–16.3 % during 2020s, 2050s and 2080s, respectively. The increased evapotranspiration demands resulted in more irrigation demand. The irrigation water allocated by the model for wheat considerably increased during future period, whereas it slightly decreased during the same period for rice cultivation. The irrigation water allocated for wheat is likely to increase by 17.0–28.0, 28.3–36.5 and 38.4–45.3 % during 2020s, 2050s and 2080s, respectively under the projected climate change scenarios. Increased irrigation requirement relates with the decreased rainfall of December, February and March during which wheat is grown, and higher temperature which resulted in increased actual evapotranspiration. Seasonal decrease in rainfall varied in the range of 5.1–26.4, 1.9–15.4 and 7.6–16.7 % during 2020s, 2050s and 2080s, respectively. This decrease in rainfall during wheat growing season is more in downstream basins as compared to upstream basin; whereas increase in temperature is higher at upstream basins as compared to downstream basins (Fig. 8). Greater increase in mean temperature at the upstream basin as compared to the downstream basin, might have greater impact on wheat yield in the upstream basin resulting in higher increase in downstream wheat yield than upstream wheat yield. The increased irrigation water allocation might have compensated for the temperature and other stresses, and resulting in increase in wheat production in the basin.

### Limitations

The SWAT model considers daily mean temperature for simulating temperature stress on crop growth and yield. For the simulation of rice and wheat we gave approximately the same date of management operation such as planting and harvesting for all HRUs due to non- availability of HRU wise data of crop wise management practices. This may not be the actual case in the entire Gomti River basin. Moreover, we considered the changes in temperature and rainfall only for the future climate change scenarios. Other factors such as radiation may affect the future rice and wheat growth and yield. In addition, the agronomic practices, technological development, land use and land cover and cropping pattern were also assumed to remain same in future. However, this simulation study provided valuable information on possible impact of climate change on rice and wheat production, irrigation requirement and change of evapotranspiration which may be useful in future planning for crop production in the Gomti River basin.

## Conclusion

This study analysed the rainfall and temperature changes during the rice and wheat growing periods, and resultant impacts on rice and wheat production and their irrigation requirements in the Gomti River basin in India. For simulation of crop production as well as basin hydrology, SWAT hydrological model was used. The MIROC (3.2, HiRes) GCM projections for A1b, B1 and A2 emission scenario were used for generation of climate change for future time periods of 2020s, 2050s and 2080s. The SWAT model performed reasonably well for the simulation of streamflow and also for the simulation of rice and wheat yield during the calibration and validation periods. This modelling study revealed increase in mean rainfall during rice growing season (*Kharif*) in the range of 7.3–17.8, 9.9–14 and 20.9–33.2 % during 2020s, 2050s and 2080s, respectively. However, during wheat growing season rainfall was projected to decrease in the range of 5.1–26.4, 1.9–15.4 and 7.6–16.7 % during 2020s, 2050s and 2080s, respectively. The simulation results showed increase in rice and wheat yield in future periods under the MIROC3.2 GCM projected climate change scenarios, provided that other factors of crop growth are favorable. Considering the entire river basin, and the three emission scenarios (A1b, B1, and A2), mean rice yield is projected to increase by 5.5–6.7, 16.6–20.2 and 26–33.4 % during the time period 2020s, 2050s and 2080s, respectively and the mean wheat yield is likely to increase by 13.9–15.4, 23.6–25.6 and 25.2–27.9 % for the same future period. Simulation results also showed increase in irrigation water allocation for wheat in the range of 17–28, 28.3–36.5 and 38.4–45.3 % during 2020s, 2050s and 2080s, respectively. In contrast, irrigation water allocation for rice was simulated to decrease, though this decrease remained <5 % during the same future time periods. These results indicates the need to improve its irrigation facilities in the basin to cope up with the decreasing rainfall for the growing of *Rabi* crops. The results of this study provide valuable information for developing adaptation plan as well for the restoration plan of the Gomti River basin.
